# Efficacy and Safety of Letibotulinumtoxin A in the Treatment of Glabellar Lines: A Randomized, Double-Blind, Multicenter, Placebo-Controlled Phase 3 Study

**DOI:** 10.1093/asj/sjac019

**Published:** 2022-02-02

**Authors:** Daniel S Mueller, Valentina Prinz, Jeffrey Adelglass, Sue Ellen Cox, Michael H Gold, Joely Kaufman-Janette, Mark S Nestor, Susan Taylor, Konstantin Frank

**Affiliations:** Tennessee Clinical Research Center, Nashville, TN, USA; Center for Clinical and Cosmetic Research, Aventura, FL, USA; Perelman Center for Advanced Medicine, University of Pennsylvania, Philadelphia, PA, USA

## Abstract

**Background:**

Letibotulinumtoxin A (Hugel, Inc., Chuncheon, Republic of Korea and CROMA Pharma, Leobendorf, Austria) is a newly manufactured neurotoxin derived from *Clostridium botulinum* strain CBFC26.

**Objectives:**

The aim of this study was to assess the efficacy and safety of letibotulinumtoxin A in reducing glabellar line severity (GLS) and to evaluate long-term safety and efficacy following repeated injections.

**Methods:**

In this prospective, randomized, parallel-group, double-blind, multicentre, placebo-controlled Phase III clinical trial, 355 subjects with moderate to severe glabella frown lines received injections of 20 U of letibotulinumtoxin A or placebo. GLS, onset and duration of effect, time to retreatment, and adverse events were evaluated. Response to treatment was defined as a GLS score of 0 or 1 (assessed by the subject and the investigator) and an improvement at Week 4 of ≥2 points in GLS score relative to baseline.

**Results:**

At 4 weeks, 78.6% of the active treatment subjects were responders based on the investigator’s assessment and 68.8% based on the subject’s assessment, resulting in a composite responder rate of 64.7% for the active treatment group, whereas the corresponding rate was 0.0% in the placebo group (*P* < 0.001). Subjects noted a substantial improvement in GL severity as early as Day 2, with the median time to onset of effect being 3 days. The mean [standard deviation] time until first retreatment for the letibotulinumtoxin A group was 127.26 [65.6] days. Letibotulinumtoxin A was well tolerated.

**Conclusions:**

Letibotulinumtoxin A demonstrates high efficacy and a convincing safety profile in the treatment of glabellar lines.

**Level of Evidence: 2:**

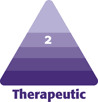

Injection of botulinum toxin is the most commonly performed nonsurgical cosmetic procedure worldwide in both women and men. According to The Aesthetic Society a total of 2,643,366 neurotoxin injections were performed in the United States in 2019.^1^ Over recent years, there has been a surge in demand for cosmetic noninvasive and minimally invasive procedures, propelled by an increasing awareness of facial beauty, the aging face, and skincare in general. This is reflected by a 35.5% increase of neurotoxin injections between 2015 and 2019.^1^ The rise in demand has been met by a growing number of manufacturers launching new products.^2,3^

The appearance of accentuated vertical glabellar lines, caused by activity of the procerus and bilateral corrugator muscles, can cause a depressed, angry, or fatigued look. Recent findings have shown that the muscular activity of the procerus and corrugator muscles increases with age,^4^ reinforcing the idea that the formation of both dynamic and static glabellar folds is caused by hyperactivity of these muscles. Injecting neurotoxins can ameliorate or even eliminate vertical glabellar lines. Furthermore, the corrugator and procerus muscles are responsible for depression of the medial- to mid-portions of the eyebrow, thus an elevation of the eyebrow can be achieved by decreasing the activity of these muscles with neurotoxins.

Letibotulinumtoxin A is a newly manufactured product derived from *Clostridium botulinum* strain CBFC26. The safety and efficacy of this neurotoxin in the treatment of noncosmetic indications, such as poststroke upper-limb spasticity and dynamic equinus foot deformity in children with cerebral palsy, has previously been established.^5,6^ Furthermore, letibotulinumtoxin A was found to be as effective as onabotulinumtoxin A in the treatment of glabellar lines and crow’s feet.^7,8^

The objective of this placebo-controlled study was to assess the efficacy and safety of letibotulinumtoxin A in reducing the severity of glabellar frown lines. In addition, we aimed to evaluate the long-term safety and efficacy following repeated injections in a subsequent open-label extension phase.

## METHODS

### Trial Design

This investigation was a prospective, randomized, parallel-group, double-blind, multicentre, placebo-controlled study comprising 2 parts. The first treatment cycle aimed to demonstrate the efficacy and safety of letibotulinumtoxin A compared with placebo. The second treatment cycle was an open-label extension phase to evaluate both efficacy after repeat injections and long-term safety. The study was conducted at 7 study centres in the United States and Europe between May 2019 and December 2020 (Yuvell Home of Aesthetics, Vienna, Austria; Research Your Health, Skintastic Medical, Plano, TX; Aesthetic Solutions PA, Chapel Hill, NC; Tennessee Clinical Research Center, Nashville, TN; Skin Research Institute LLC, Coral Gables, FL; Center for Clinical and Cosmetic Research, Aventura, FL; Perelman Center for Advanced Medicine–University of Pennsylvania, Philadelphia, PA). Ethical approval was granted by a central IRB (Copernicus, Cary, NC; IRB number 20190353). In addition, the US FDA, the Austrian independent ethics committee (Medizinische Universität Wien), and a competent authority also approved the protocol prior to its initiation. This study was performed in adherence with the Declaration of Helsinki, Good Clinical Practice, the Code of Federal Regulation (Title 21, Part 312), and local regulatory requirements. The ClinicalTrials.gov identifier is NCT03985982. Written consent was obtained from the patients, who thereby agreed to the use and analysis of their data.

### Participants

Eligibility criteria for participants are given in Table 1. Eligible subjects were randomly allocated at baseline (3:1 randomization scheme) to the active treatment or placebo group in the first treatment cycle. Randomization was performed via an interactive web response system at each study site and each subject received a unique randomization code.

**Table 1.** Detailed Inclusion and Exclusion Criteria

Inclusion criteria
Subjects who meet all the following criteria are eligible for this study:
• Aged ≥18 years or older at the time of screening (upper limit 75 years, inclusive)
• Has moderate to severe glabellar frown lines at maximum frown (GLS score of 2 or 3) as determined by in-clinic assessments by both the investigator and the subject (where 0 = none, 1= mild, 2 = moderate, 3 = severe)
• Subject has a stable medical condition with no uncontrolled systemic disease
• Female subjects of childbearing potential must test negative for pregnancy and agree to use highly effective birth control during the course of the study
• Subjects who wear glasses must be able to adequately self-assess the severity of their glabellar lines (according to the GLS), without glasses obstructing the forehead area
• Moderate to severe glabellar lines have an important psychological impact on the subject, as indicated by scores >0 on either the Emotional or the Social Functioning subscale of the modified Skindex-16 (GL-QoL).
Exclusion criteria
Subjects who meet any of the following criteria are not eligible for this study:
• Previous treatment with any serotype of botulinum toxin for any indication within the 12 months prior to screening, or any planned treatment with botulinum toxin of any serotype for any reason during the trial (other than the investigational treatment)
• Known hypersensitivity to the study medication or its excipients
• Any medical condition that may place the subject at increased risk due to exposure to botulinum toxin, including diagnosed myasthenia gravis, Eaton-Lambert syndrome, amyotrophic lateral sclerosis, profound atrophy or weakness in the target muscles, or any other condition (at the investigator's discretion) that might interfere with neuromuscular function or contraindicate botulinum toxin therapy.
• Facial laser or light treatment, microdermabrasion, superficial peels, or retinoid therapy within the 3 months prior to screening or planned during the study
• Apart from the procedures specified above, previous treatment with any facial aesthetic procedure in the glabellar area (including chemical peeling, injection with biodegradable fillers) within 12 months prior to screening or planned during the study
• Previous insertion of permanent material in the glabellar area or planned during the study.
Any surgery, or history of surgery, in the glabellar area including surgical removal of the corrugator, procerus, or depressor supercili muscles or a combination of these, or scars in the glabellar area, or such surgery planned during the study
• Active skin disease/infection or irritation at the treatment area
• Inability to substantially lessen glabellar frown lines even by physically spreading them apart
• Use of a muscle relaxant within 2 weeks prior to screening or planned during the study
Marked facial asymmetry or ptosis of eyelid and/or eyebrow, or current facial palsy or neuromuscular junction disorders as judged by the investigator
• Pregnant, breastfeeding, or planning to become pregnant during the trial
• Use of prohibited medication including anticholinergic drugs, or drugs which could interfere with neuromuscular function, including aminoglycoside antibiotics and curare-like compounds, within 2 weeks prior to screening or planned during the study
• Planned surgery with general anesthetic (use of local anesthetic outside the glabellar area is permitted)
• Participation in another clinical study within 1 month of screening and throughout the trial
Previous participation in another botulinum toxin aesthetic study which involved the treatment of glabellar lines in combination with canthal lines and/or forehead lines in the previous 18 months
• Chronic drug or alcohol abuse (as per investigator discretion)

GL-QoL, glabellar line quality of life score; GLS, glabellar line severity.

### Intervention

**Figure 1.**
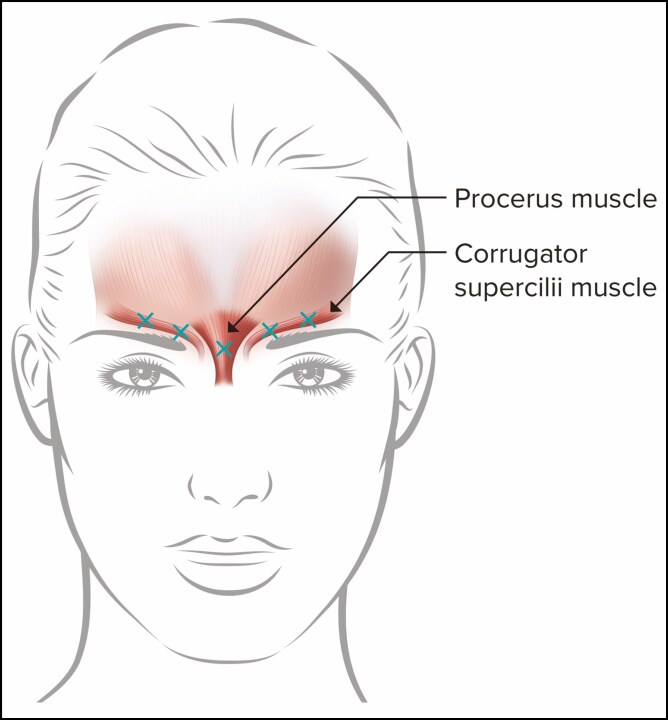
Schematic drawing depicting the injection sites: 2 injections in each corrugator supercilii muscle and 1 injection in the procerus muscle were administered.

Investigators and subjects were blinded to the treatment administered for the first (placebo-controlled) treatment cycle. Active treatment vials, containing 50 U of letibotulinumtoxin A, or empty placebo vials, were reconstituted with 1.25 mL sterile physiological saline by an unblinded investigator, not involved in any study data collection activities. Patients assigned to the letibotulinumtoxin A group received a total of 20 U active letibotulinumtoxin A; this comprised injecting 4 U (0.1 mL) into each of the 5 intramuscular injection sites: 2 injections in each corrugator supercilii muscle and 1 injection in the procerus muscle (Figure 1). Injection in the corrugator supercilii muscle was performed immediately above the medial margin of the eyebrows and for the second injection point approximately 1 cm above the supraorbital ridge. The injection site of the procerus muscle was just above the midline of the nasal bridge where horizontal wrinkles are made between the medial end of the eyebrows. The same injection points were targeted in patients assigned to the placebo group, who were injected with an equal volume of sterile physiological saline solution.

Subjects received a maximum of 4 treatment cycles over the duration of the study, a single treatment in the first cycle compared with placebo, and up to 3 subsequent treatments in the open-label extension phase. The first treatment cycle lasted at least 12 weeks and ended when the subject qualified for retreatment. All subjects could enter the open-label extension phase and were dosed with letibotulinumtoxin A (20 U); the same injection pattern and volume were used for subsequent retreatments. Evaluation for retreatment took place at the earliest at 12 weeks after the first/previous treatment, according to predefined retreatment criteria (Table 2). Subjects who did not qualify for retreatment at Week 12 were offered retreatment at a later visit (at 4-weekly intervals thereafter) up until 48 weeks after the start of the study. Maximum study duration per participant was up to 62 weeks. A maximum of 3 treatments in the open-label extension phase were performed.

**Table 2.** Eligibility Criteria for Retreatment

Retreatment criteria
• At time of retreatment subject does not have relevant changes to their health status from enrollment, which would have prevented subject’s entry into the study according to the inclusion and exclusion criteria
• The subject must have been randomly assigned to receive treatment and must have received at least 1 treatment (letibotulinumtoxin A or placebo)
• A minimum of 12 weeks must have elapsed since the previous study treatment
• The subject's glabellar lines at maximum frown must have relapsed to a GLS score of 2 or 3 as determined by both the investigator and the subject
• No relevant infection or inflammation in the planned injection area
• Negative urine pregnancy test in women of child-bearing potential
• The subject must have received fewer than 4 study treatments
• The subject must agree and consent to retreatment
• Retreatment will be performed at the latest by Week 48

GLS, glabellar line severity.

### Assessments and Outcomes

After the first treatment (letibotulinumtoxin A or placebo), subjects attended follow-up visits at 1, 2, and 4 weeks, and at 4-weekly intervals thereafter for evaluation of efficacy and safety. After the retreatment, subjects attended follow-up visits after 1 and 4 weeks and at 4-weekly intervals thereafter. At Week 2 and Week 8 of each open-label cycle a telephone visit was conducted.

The following assessments were performed:

- Glabellar line severity score (GLS):^9^ both the subject and the investigator determined the GLS at baseline, at study visits at 1, 2, 4, 8, and 12 weeks and at 4-weekly intervals thereafter, until the subject qualified for retreatment. In the open-label extension phase, GLS was evaluated at Weeks 1 and 4 after retreatment and at 4-weekly intervals thereafter. Responders to active treatment or placebo were defined as subjects with a GLS score of 0 or 1 and an improvement of ≥2 points in GLS score (at maximum frown) relative to baseline.
- In addition, onset of effect was assessed by subjects daily for the first 2 weeks after treatment, with their findings being recorded in a diary.

### Primary Endpoint

The primary efficacy endpoint was the proportion of responders among the active and placebo group, based on both the investigator’s and the subject’s in-clinic assessments at Week 4 of the first treatment cycle. Thus, the primary endpoint was a composite endpoint comprising investigator and subject assessments of treatment effectiveness to adhere to local regulatory requirements.

### Secondary Endpoints

The following secondary endpoints were defined:

- Responder rate relative to baseline, based on both the investigator’s and the subject’s in-clinic assessment at Weeks 1, 2, 8, 12, and 16 for the active treatment and placebo group;
- Number of subjects in the active treatment group with severe glabellar lines at baseline and no lines at Week 4;
- Proportion of subjects of the active treatment group with a ≥1-point reduction at maximum frown in GLS based on both the investigator’s and the subject’s in-clinic assessment at Weeks 1, 2, 4, 8, and 16;
- Median time to onset of effect of ≥1-point improvement in GLS from baseline based on subject’s diary data for the active treatment group;
- Number of subjects in the active treatment group reporting a ≥1-point reduction in GLS at maximum frown based on subject diary data at Days 0, 1, 2, and 3;
- Duration of treatment, defined as time point where a statistical significance of responder rate (as defined in the primary endpoint) still existed between active treatment group and placebo;
- Mean duration until retreatment of the active group in the first treatment cycle.

### Safety

Safety was evaluated by assessing the frequency, severity, and causal relationship of adverse events (AEs), serious AEs, and AEs of special interest during the entire study period. Serum samples were tested for the presence of antibodies to botulinum toxin with an anti-drug antibody ELISA assay at screening, at the Week 4 visit, and at the end of cycle visit for each treatment (screening assay; BIAcore T200 surface plasmon resonance assay). If the screening assay was positive, a confirmation assay was performed (confirmation assay; BIAcore T200 surface plasmon resonance assay). If the confirmation assay was positive, antibody titer and detection of neutralizing antibodies was performed. Safety assessments were completed by evaluating laboratory tests (hematology, clinical chemistry), monitoring of vital signs, and electrocardiograms.

### Sample Size Calculation

A sample size of 225 subjects in the letibotulinumtoxin A group was calculated, assuming a response rate of 60% in the letibotulinumtoxin A group, a 2-sided 95% CI, and a distance from the CI limit of approximately 5.6%. A 3:1 randomization of letibotulinumtoxin A (225 subjects) to placebo (75 subjects) was proposed, which was considered to be adequate for a precise estimate of responder rate.

### Statistical Analyses

All statistical procedures were performed with Statistical Analysis Software (SAS) version 9.4. The statistical analyses were performed after all subjects completed the re-evaluation for the retreatment visit at Week 16 of the first treatment cycle or completed the double-blind phase (whichever occurred earlier). Two-sided 95% CIs were provided when relevant. The statistical tests for the primary endpoint and key secondary endpoints were 1-sided and applied a significance (α) level of 0.025. Continuous variables were summarized by descriptive statistics, including number of subjects (n), mean, standard deviation (SD), and median, minimum, and maximum. For categoric variables, summaries included counts of subjects and percentages in the corresponding categories. The primary endpoint was analyzed with the Cochran-Mantel-Haenszel test. Study center was used as a stratification variable and small study centers with  <3 placebo subjects were combined. The full analysis set (FAS) population was used to evaluate the efficacy assessments. The safety analysis set was used to evaluate the safety assessments.

## RESULTS

### Subject Disposition and Demographics

**Figure 2.**
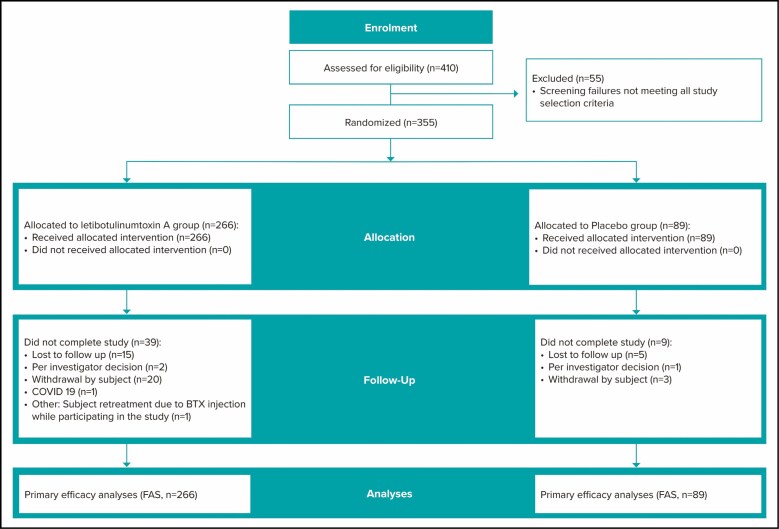
CONSORT flow diagram. The FAS population included all randomized subjects, regardless of whether they received study medication. Within the FAS population, a subject was considered for the treatment assigned by randomization rather than the treatment actually received, if different, ie, following the intention-to-treat principle. The FAS population was used to evaluate the efficacy assessments. FAS, full analysis set.

A total of 355 subjects, of whom 328 were females (92.4%), with a mean [SD] age of 51.5 [11.6] years (range, 21.0-75.0 years) and a mean BMI of 26.46 [5.1] kg/m^2^ (range, 15.1-55.8 kg/m^2^) were randomly allocated to receive treatment. Fitzpatrick skin type I, II, III, IV, V, or VI was reported for 10 (2.8%), 131 (36.9%), 108 (30.4%), 67 (18.9%), 27 (7.6%), and 12 (3.4%) subjects, respectively. At baseline in the active treatment group, 64 patients had moderate, and 202 patients had severe lines according to in-clinic assessment by the investigators, whereas in the active treatment group 70 subjects had moderate and 196 subjects had severe lines according to the in-clinic assessment. Further demographic variables are summarized in Table 3. Of the 355 randomized subjects, 266 were randomly assigned to the letibotulinumtoxin A group and 89 subjects were randomly assigned to the placebo group (FAS and SAF). The primary reasons for discontinuation from the double-blind phase were withdrawal by subject (23 subjects) and loss to follow-up (20 subjects) (Figure 2 and Appendix, available online at www.aestheticsurgeryjournal.com).

**Table 3.** Demographics of the Subjects Enrolled in the Double-Blind Phase, Open-Label Phase, and Overall

		Double-blind		Open-label	Overall (N = 355)
		Letibotulinumtoxin A (N = 266)	Placebo (N = 89)	Letibotulinumtoxin A (N = 323)	
Age, years	Mean [SD]	52.2 [11.24]	49.4 [12.36]	51.7 [11.55]	51.5 [11.58]
Sex, n (%)	Male	18 (6.8)	9 (10.1)	24 (7.4)	27 (7.6)
	Female	248 (93.2)	80 (89.9)	299 (92.6)	328 (92.4)
Race, n (%)	Caucasian	236 (88.7)	79 (88.8)	290 (89.8)	315 (88.7)
	African American	25 (9.4)	7 (7.9)	25 (7.7)	32 (9.0)
	Asian	4 (1.5)	1 (1.1)	5 (1.5)	5 (1.4)
	Other	1 (0.4)	2 (2.2)	3 (0.9)	3 (0.8)
BMI, kg/m^2^	Mean [SD]	26.34 [5.391]	26.84 [4.224]	26.29 [5.000]	26.46 [5.122]
Fitzpatrick skin type, n (%)	Type I	7 (2.6)	3 (3.4)	10 (3.1)	10 (2.8)
	Type II	107 (40.2)	24 (27.0)	123 (38.1)	131 (36.9)
	Type III	75 (28.2)	33 (37.1)	100 (31.0)	108 (30.4)
	Type IV	49 (18.4)	18 (20.2)	59 (18.3)	67 (18.9)
	Type V	18 (6.8)	9 (10.1)	21 (6.5)	27 (7.6)
	Type VI	10 (3.8)	2 (2.2)	10 (3.1)	12 (3.4)

SD, standard deviation.

### Primary Endpoint

**Figure 3.**
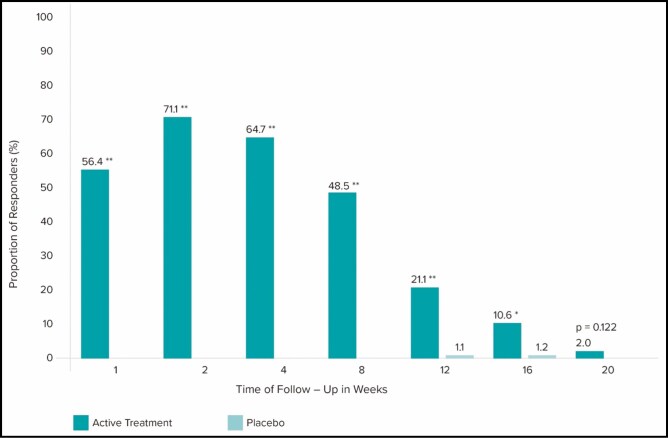
Bar graph depicting the proportion of responders as a percentage based on composite assessment at Weeks 1, 2, 4, 8, 12, 16, and 20 for the active treatment and placebo groups. Responders to active treatment or placebo were defined as subjects with a GLS score of 0 or 1 and an improvement of ≥2 points in GLS score (at maximum frown) relative to baseline. *P = 0.005, **P < 0.001. GFS, glabellar line severity.

The composite responder rate (at maximum frown) at Week 4 from baseline was 64.7% for the active treatment group and 0.0% for the placebo group (*P* < 0.001). The responder rates as rated by the subject were lower than those rated by the investigator, but still showed a large treatment effect 4 weeks after baseline. In the investigator assessment, the responder rate was 78.6% for the letibotulinumtoxin A group vs 68.8% for the subject assessment (Figure 3).

### Secondary Endpoints

Composite responder rates of the subjects undergoing active treatment were 56.4%, 71.1%, 48.5%, 21.1%, 10.6% and 2.0% at Weeks 1, 2, 8, 12, 16, and 20 for the active treatment group, whereas responder rates in the placebo group were 0.0%, 0.0%, 0.0%, 1.1%, 1.2%, and 0.0% at Weeks 1, 2, 8, 12, 16, and 20 with *P* ≤ 0.005 until Week 16 and *P* = 0.122 at Week 20 (Figure 3). A total of 98 subjects (48.5%) who initially presented with severe lines at baseline had no lines at Week 4.

**Figure 4.**
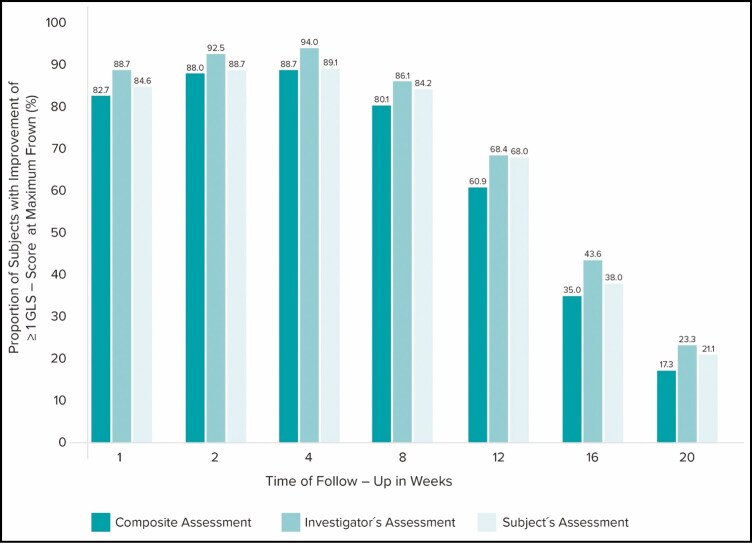
Proportion of subjects as a percentage with a ≥1-point improvement in GLS score at maximum frown based on composite assessment, investigator’s assessment and subject’s assessment at Weeks 1, 2, 4, 8, 12, 16, and 20.

The proportion of subjects presenting with a  ≥1-point reduction in GLS at maximum frown based on both investigator and subject in-clinic assessment was 82.7%, 88.0%, 88.7%, 80.1%, 60.9%, 35.0%, and 17.3% at Weeks 1, 2, 4, 8, 12, 16, and 20, respectively (Figure 4). Based on the subjects’ diary data the median time to onset of a  ≥1-point improvement in GLS from baseline was 3.0 days in the letibotulinumtoxin A group. A total of 24.0% of the subjects treated with letibotulinumtoxin A reported a  ≥1-point reduction within 24 hours (Day 0), whereas 44.5%, 63.9%, and 74.5% reported a ≥1-point reduction at Days 1, 2, and 3, respectively. The duration of treatment, defined as the time point at which a statistically significant difference of responder rate still existed between the active treatment group and placebo, was 16 weeks. Time until retreatment for the active treatment group was on average 127.26 [65.6] days in the double-blind phase.

### Safety Assessments

There were no serious AEs related to study drug, and no treatment-emergent adverse events (TEAEs) that led to study discontinuation. No deaths were reported during the study. During the double-blind phase 48 (18.0%) subjects within the active treatment and 14 (15.7%) subjects within the placebo group reported TEAEs. During the double-blind phase, headache (with or without relation to the study drug or the injection procedure) was the only TEAE with an incidence of ≥1% of subjects in the botulinum neurotoxin type A drug product (BoNT/A-DP) group that was reported more frequently in the BoNT/A-DP group than in the placebo group (10 [3.8%] subjects in the BoNT/A-DP group vs 2 [2.2%] subjects in the placebo group). In the active treatment group, 4 subjects (1.5%) reported TEAEs related to study medication and injection procedure. One subject reported a severe TEAE, which was not related to study drug or injection procedure. No TEAE led to discontinuation. In the open-label phase a total of 80 (24.8%) subjects reported any TEAE, of which 8 (2.5%) were drug related and 6 (1.9%) were related to the injection procedure. Three (0.9%) TEAEs were severe; however, none were related to the study drug or injection procedure. None of the TEAEs led to discontinuation (Table 4).

**Table 4.** Overall Summary of Treatment-Emergent Adverse Events in the Open-Label Phase, Both Phases, and Overall

	Double-blind		Open-label	Both phases	Overall
	Letibotulinumtoxin A (N = 266)	Placebo (N = 89)	Letibotulinumtoxin A total (N = 323)	Letibotulinumtoxin A total (N = 350)	(N = 355)
Overall incidence	n (%)	n (%)	n (%)	n (%)	n (%)
Subjects with any TEAE	48 (18.0)	14 (15.7)	80 (24.8)	118 (33.7)	127 (35.8)
Study drug-related TEAE	4 (1.5)	0 (0.0)	8 (2.5)	12 (3.4)	12 (3.4)
Injection procedure–related TEAE	4 (1.5)	0 (0.0)	6 (1.9)	10 (2.9)	10 (2.8)
Severe TEAE	1 (0.4)	0 (0.0)	3 (0.9)	4 (1.1)	4 (1.1)
Severe study drug-related TEAE	0 (0.0)	0 (0.0)	0 (0.0)	0 (0.0)	0 (0.0)
Severe injection procedure–related TEAE	0 (0.0)	0 (0.0)	0 (0.0)	0 (0.0)	0 (0.0)
Any SAE	1 (0.4)	1 (1.1)	4 (1.2)	5 (1.4)	6 (1.7)
Any TEAE leading to discontinuation	0 (0.0)	0 (0.0)	0 (0.0)	0 (0.0)	0 (0.0)
Study medication related TEAE leading to discontinuation	0 (0.0)	0 (0.0)	0 (0.0)	0 (0.0)	0 (0.0)
TEAEs leading to death	0 (0.0)	0 (0.0)	0 (0.0)	0 (0.0)	0 (0.0)

SAE, serious adverse event; TEAE, treatment-emergent adverse event.

At baseline, 27 subjects (23 from the active treatment group and 4 from the placebo group) received a reactive anti-drug antibody screening test. No reactivity was detected at the confirmatory assay test. Thus, no neutralizing activity or titer was assessed. During the double-blind phase and open-label phase of the study, descriptive statistics showed only small and not clinically relevant changes from baseline to Week 4 for each treatment cycle in the clinical chemistry and hematology parameters for both the letibotulinumtoxin A and placebo groups. The numbers of treatment-related and injection-procedure-related AEs are given in Tables 5 and 6.

**Table 5.** Number of Treatment-Related Adverse Events by System Organ Class and Preferred Term for the Double-blind, Open-Label, and Both Phases

	Double-blind		Open-label	Both phases
System organ class preferred term	Letibotulinumtoxin A (N = 266)	Placebo (N = 89)	Letibotulinumtoxin A (N = 323)	Letibotulinumtoxin A (N = 350)
	n (%)	n (%)	n (%)	n (%)
Subjects with any study drug-related TEAE	4 (1.5)	0 (0.0)	8 (2.5)	12 (3.4)
Nervous system disorders	3 (1.1)	0 (0.0)	3 (0.9)	6 (1.7)
Headache	3 (1.1)	0 (0.0)	3 (0.9)	6 (1.7)
Eye disorders	0 (0.0)	0 (0.0)	3 (0.9)	3 (0.9)
Eyelid ptosis	0 (0.0)	0 (0.0)	3 (0.9)	3 (0.9)
Diplopia	0 (0.0)	0 (0.0)	1 (0.3)	1 (0.3)
Injury, poisoning, and procedural complications	1 (0.4)	0 (0.0)	1 (0.3)	2 (0.6)
Contusion	0 (0.0)	0 (0.0)	1 (0.3)	1 (0.3)
Procedural headache	1 (0.4)	0 (0.0)	0 (0.0)	1 (0.3)
Skin and subcutaneous tissue disorders	0 (0.0)	0 (0.0)	1 (0.3)	1 (0.3)
Brow ptosis	0 (0.0)	0 (0.0)	1 (0.3)	1 (0.3)

TEAE, treatment-emergent adverse event.

**Table 6.** Number of Injection Procedure-Related Adverse Events by System Organ Class and Preferred Term for the Double-blind, Open-Label, and Both Phases

	Double-blind		Open-label	Both phases
System organ class preferred term	Letibotulinumtoxin A (N = 266)	Placebo (N = 89)	Letibotulinumtoxin A (N = 323)	Letibotulinumtoxin A (N = 350)
	n (%)	n (%)	n (%)	n (%)
Subjects with any injection procedure–related TEAEs	4 (1.5)	0 (0.0)	6 (1.9)	10 (2.9)
Nervous system disorders	3 (1.1)	0 (0.0)	1 (0.3)	4(1.1)
Headache	3 (1.1)	0 (0.0)	1 (0.3)	4(1.1)
Eye disorders	0 (0.0)	0 (0.0)	3 (0.9)	3 (0.8)
Eyelid ptosis	0 (0.0)	0 (0.0)	2 (0.6)	2 (0.6)
Diplopia	0 (0.0)	0 (0.0)	1 (0.3)	1 (0.3)
Eye pain	0 (0.0)	0 (0.0)	1 (0.3)	1 (0.3)
Injury, poisoning, and procedural complications	2 (0.8)	0 (0.0)	1 (0.3)	3 (0.8)
Contusion	1 (0.4)	0 (0.0)	1 (0.3)	2 (0.6)
Procedural headache	1 (0.4)	0 (0.0)	0 (0.0)	1 (0.3)
Skin and subcutaneous tissue disorders	0 (0.0)	0 (0.0)	1 (0.3)	1 (0.3)
Acne	0 (0.0)	0 (0.0)	1 (0.3)	1 (0.3)
Skin swelling	0 (0.0)	0 (0.0)	1 (0.3)	1 (0.3)

TEAE, treatment-emergent adverse event.

## DISCUSSION

**Figure 5.**
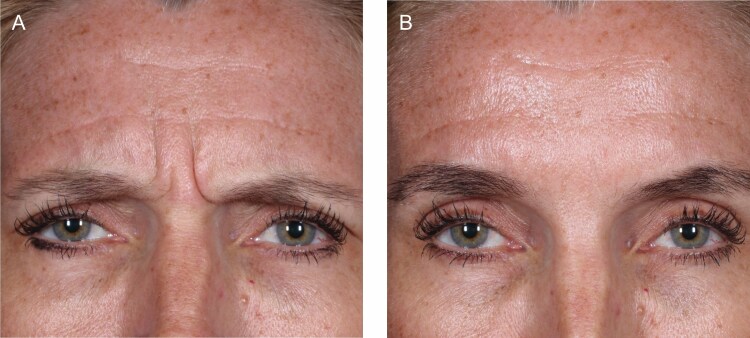
Before-and-after images of a 47-year-old female subject at (A) baseline and (B) after 4 weeks.

**Figure 6.**
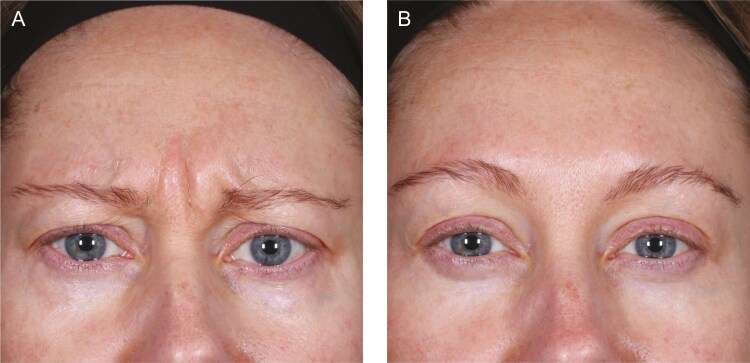
Before-and-after images of a 38-year-old female subject at (A) baseline and (B) after 4 weeks.

This prospective, randomized, parallel-group, double-blind, multicentre, placebo-controlled Phase III study, followed by an open-label extension, demonstrates high efficacy and a convincing safety profile of letibotulinumtoxin A in the treatment of glabellar lines in a study population of 355 subjects (Figures 5 and 6). Overall, the difference in the composite responder rates between the letibotulinumtoxin A and placebo groups at Week 4 after baseline was 64.66% in favor of the active treatment, thereby highlighting its superiority. These rates are comparable to the outcome of previous high-quality studies that investigated the efficacy of neurotoxins such as prabotulinumtoxinA, onabotulinumtoxinA, daxibotulinumtoxinA, or abobotulinumtoxinA injected according to a similar protocol.^10-14^ A randomized controlled trial (RCT) investigating prabotulinumtoxinA vs onabotulinumtoxinA for the treatment of glabellar lines reported response rates of up to 87.2% and 82.8% compared with placebo, respectively.^13^ In comparison, these studies defined responders as patients with a GLS score of 0 or 1 at maximum frown on Day 30 by investigator assessment only. In the presented trial, the definition of responders was narrower and more specific, being composed of a composite endpoint including also the subject’s GLS assessment in addition to an improvement of ≥2 points in the GLS score (at maximum frown) relative to baseline, and thus adhering to the recommendations of the FDA guidance for industry in developing botulinum toxin drug products.^15^ To ensure clinical significance, the FDA requires treatment success to be defined as achievement of a score of 0 or 1 in addition to a 2-grade improvement, on both the investigator’s scale and the subject’s self-assessment scale, compared with baseline.^15^ The differences in the definition of responders could thus account for the slightly lower response rates found for letibotulinumtoxin A in this study. For example, when considering only the investigator assessment, the responder rate increased up to 78.6% for the letibotulinumtoxin A group, highlighting the impact the composite endpoint might have on the assessment of treatment efficacy. Moreover, the aforementioned RCT reported a responder rate of 4.4% for the placebo group, compared with the 0.0% in the composite endpoint of the presented study, possibly pointing towards a tendency to overreport response rates. Importantly, a RCT investigating only the efficacy and safety of prabotulinumtoxinA reported response rates of 67.5% and 70.4% in 2 identical Phase III clinical trials, matching the data presented in this manuscript.^16^ Similarly, 2 Phase III RCTs investigating the efficacy of daxibotulinumtoxinA in subjects with moderate or severe glabellar lines revealed response rates of 73.6% and 74.0% for the neurotoxin, with responders being defined as showing a ≥2-point improvement in GLS at maximum frown at Week 4 according to both investigator and subject ratings.^11^

The mean time period until retreatment was about 4 months (19 weeks) in the first treatment cycle, which is satisfactory and in line with most common retreatment schemes performed by practitioners. In the letibotulinumtoxin A group, the median time to onset of a ≥1-point improvement in GLS from baseline was 8.0 days for both investigator and subject assessments. However, this needs to be considered with caution as this was the first follow-up after treatment. Based on the subject diaries a total of 24.0% reported a ≥1-point improvement within the first 24 hours and 74.5% at Day 3, which rather reflects the time of onset of letibotulinumtoxin A.

Neutralizing antibodies can cause a loss of target muscle response to the paralyzing drug due to immunoresistance against the neurotoxin; ^17^ however, no such antibodies were detected in this study even after repeat treatments. This study has shown that the safety profile of letibotulinumtoxin A, even when injected repeatedly, can be considered excellent. There were 2.3% more TEAEs in the active group than in the placebo group during the double-blind phase of the study and no serious side effects related to the study drug were reported. Furthermore, the incidence of paralysis of the levator palpaebrae muscle was reported to be 0.0% in the double-blind phase. An incidence of 3 treatment-related cases of eyelid ptosis and an incidence of 2 injection-related cases of eyelid ptosis (0.9% and 0.6%. respectively) were reported in the open-label phase and 0.6% in the open-label phase, which is in line with recent literature.^18^

This study is not free of limitations. Although the gold standard for the assessment of the severity of vertical glabellar lines is assessment based on validated photographic numeric scales, novel techniques such as 3-dimensional surface imaging with vectoral skin analysis or surface electromyography are also modalities that can be utilized to assess the efficacy of neurotoxins. However, these techniques are still not evolved and properly validated and are not ready for a multicenter study with a large number of participants that needs to be approved by regulatory authorities. Another limitation of this investigation is the fact that fewer males (6.8%) than females participated. However, most patients who seek treatment with neurotoxins for the amelioration of vertical glabellar lines are females.

## CONCLUSIONS

Letibotulinumtoxin A demonstrated a high efficacy and an excellent safety profile in the treatment of glabellar lines even after several retreatments. These findings are in line with previous investigations that have shown letibotulinumtoxin A to be comparable in efficacy to onabotulinumtoxin A for the treatment of glabellar lines; however, this investigation adds more long-term data on the efficacy and safety of letibotulinumtoxin A.

## Supplementary Material

sjac019_suppl_Supplementary_Appendix
